# Effects of 12 weeks of beta-hydroxy-beta-methylbutyrate free acid, adenosine triphosphate, or a combination on muscle mass, strength, and power in resistance trained individuals

**DOI:** 10.1186/1550-2783-10-S1-P17

**Published:** 2013-12-06

**Authors:** Ryan P Lowery, Jordan Joy, John A Rathmacher, Shawn M Baier, John C Fuller, Ralf Jäger, Stephanie M C Wilson, Jacob M Wilson

**Affiliations:** 1 Department of Health Sciences and Human Performance, The University of Tampa, Tampa, FL, USA; 2 Metabolic Technologies, Inc., Iowa State University Research Park, Ames, IA; USA; 3 Department of Animal Science, Iowa State University, Ames, IA USA; 4 Increnovo LLC, Milwaukee, WI, USA; 5 Department of Nutrition, IMG Performance Institute, IMG Academy, Bradenton, FL, USA

## Introduction

Adenosine-Triphosphate (ATP) supplementation maintains performance and increases volume under high fatiguing contractions. However, greater fatigue increases recovery demands between training sessions. Studies utilizing HMB free acid (HMB-FA) supplementation suggest that the supplement speeds regenerative capacity. However, we are unaware of studies investigating whether synergism exists between the two. Therefore, we investigated the effects of 12 weeks of HMB-FA, ATP, or a combination of the two on lean mass (LBM), strength, and power in trained individuals. We also determined these supplements effects on performance during an overreaching cycle.

## Methods

A 3-phase double-blind, placebo- and diet-controlled intervention study was conducted. Subjects were given either 3g per day of HMB in the free acid form (Metabolic Technologies, Ames, IA), 400mg per day of Peak ATP®(TSI, Missoula, MT), or a combination of the two. Phase 1 consisted of an 8-week periodized resistance-training program; Phase 2 was a 2-week overreaching cycle in which training volume and frequency increased; and Phase 3 was a 2-week taper in which training volume and frequency were decreased. Muscle mass, strength, and power were examined at weeks 0, 4, 8, and 12 to assess the chronic effects of supplementation; and assessment of these was performed at weeks 9 and 10 of the overreaching cycle.

## Results

Supplementation with ATP and HMB-FA increased strength gains over the 12-week study (ATP*time, p < 0.05 and HMB*time, p <0.05, respectively). Strength gains following training were greatest in the HMB-FA+ATP group, followed by the HMB-FA, ATP, and placebo groups respectively. No significant interaction (HMB-FA*ATP*time, p > 0.05) was observed indicating that the HMB and ATP supplementation effects were additive. During the overreaching cycle, strength declined in the placebo (-4.5%) group, but this decline was blunted in both the ATP (-2%) and HMB-FA (-.5 %) groups. Surprisingly, the HMB-FA+ATP group continued to gain strength (+1.2%). Over the 12-weeks of training vertical jump power increased to the greatest extent in the HMB+ATP group, followed by the HMB-FA, ATP, and placebo groups, respectively. The percentage increases in vertical jump power were synergistic with HMB-FA and ATP supplemented in combination (HMB-FA*ATP*time, p < 0.004). Vertical jump power during the overreaching cycle decreased more in the placebo group, 5.0±0.4%, compared with the smaller decreases in vertical jump power for the HMB-FA, ATP, and HMB-FA+ATP supplemented groups, 1.4±0.4, 2.2±0.4, and 2.2±0.5%, respectively, over weeks 9 and 10 (t-test, p < 0.05). Lean body mass was increased in an additive manner by 2.1±0.5, 7.4±0.4, 4.0±0.4, and 8.5±0.8 kg in placebo, HMB-FA, ATP, and HMB-FA+ATP-supplemented participants, respectively (t-test, p < 0.05), and fat percentage only decreased in the HMB supplemented groups.

## Conclusions

Our results suggest that HMB-FA, ATP, and the combination can enhance LBM, and strength, in an additive manner, with power increasing synergistically when HMB-FA and ATP are combined. These supplements also appear to blunt the typically overreaching response seen to high volume, low recovery training cycles.

